# On the Tuning of High-Resolution NMR Probes

**DOI:** 10.1002/cphc.201402236

**Published:** 2014-09-11

**Authors:** Maria Theresia Pöschko, Judith Schlagnitweit, Gaspard Huber, Martin Nausner, Michaela Horničáková, Hervé Desvaux, Norbert Müller

**Affiliations:** [a] Institute of Organic Chemistry, Johannes Kepler University Linz, Altenbergerstraße 69 4040 Linz (Austria), Fax: (+43) 732-2468-8747 E-mail: norbert.mueller@jku.at; [b] CEA, IRAMIS, NIMBE, Laboratoire Structure et Dynamique par Résonance Magnétique, SIS2 M UMR CEA/CNRS 3299 CEA/Saclay, 91191 Gif-sur-Yvette (France), Fax: (+33) 16908-2199 E-mail: herve.desvaux@cea.fr

**Keywords:** analytical methods, magnetic properties, NMR spectroscopy, radiation damping, spin noise

## Abstract

Three optimum conditions for the tuning of NMR probes are compared: the conventional tuning optimum, which is based on radio-frequency pulse efficiency, the spin noise tuning optimum based on the line shape of the spin noise signal, and the newly introduced frequency shift tuning optimum, which minimizes the frequency pushing effect on strong signals. The latter results if the radiation damping feedback field is not in perfect quadrature to the precessing magnetization. According to the conventional RLC (resistor–inductor–capacitor) resonant circuit model, the optima should be identical, but significant deviations are found experimentally at low temperatures, in particular on cryogenically cooled probes. The existence of different optima with respect to frequency pushing and spin noise line shape has important consequences on the nonlinearity of spin dynamics at high polarization levels and the implementation of experiments on cold probes.

## 1. Introduction

The tuning of an NMR probe is an important routine procedure, often automated and thus invisible to users of state-of-the-art NMR systems. An optimally tuned probe should deliver a maximum signal-to-noise ratio (SNR) for the observed spins, while at the same time the radio-frequency (rf) power applied for excitation is used with optimum efficiency, that is, achieving a maximum nutation angle per energy unit.[1, 2] Moreover, apart from the direct influence of tuning on the signal-to-noise ratios of NMR signals from small magnetization, there are also remarkable effects, like radiation damping and frequency pushing on the NMR response of large magnetization.[3, 4] These effects are due to the precessing magnetization inducing an electric current inside the detection coil, which in turn creates a magnetic feedback field. The amplitude and the phase of this induced feedback field depend on the difference between the electronic circuit resonance frequency *ω*_LC_ and the nuclear spin Larmor frequency *ω*_0_. At perfect tuning conditions, *ω*_LC_=*ω*_0_, the feedback field is in quadrature (meaning 90° out-of-phase) to the precessing magnetization and its contribution to the spin dynamics reduces to radiation damping, which for small excitation pulses results in a broadening of the resonance line.[3] If the tuning is not perfect the feedback field also induces a change of the observed resonance frequency of the large nuclear magnetization, called frequency pushing.[5] Further parameters to monitor when tuning an NMR probe are the spin noise line shape, previously used to attain the spin noise tuning optimum (SNTO),[6–8] and the radiation damping rate.[3]

In this paper we investigate the tuning and matching dependence of conventional and cold NMR probes with respect to the SNR, radiation damping effects and line shapes in pulse and spin noise spectra. Our results are compared to previous observations[5, 7–10] and are used to propose a new tuning target, the frequency shift tuning optimum (FSTO). At the FSTO the resonance frequency of high nuclear polarization is independent of the longitudinal magnetization, which is advantageous for suppressing solvent signals on cryogenically cooled probes. According to the frequently used resistor–inductor–capacitor (RLC) model of NMR detection circuits[11] the SNTO, and the FSTO as well as the conventional tuning optimum (CTO) should coincide. Their discrepancy has direct implications on the implementation of NMR pulse sequences in particular on cold probes and proves the inadequacy of the RLC model in many cases.

## 2. Results and Discussion

### 2.1. General Features of NMR Probe Tuning

The electronic circuit of a high-resolution NMR probe is usually represented by a coil of inductance *L* and resistance *r*, a tuning capacitor *C*_t_ in parallel to the coil and a matching capacitor *C*_m_ in series to the previous elements. In a good approximation, the first capacitor allows one to change the electronic resonance frequency *ω*_LC_ of the circuit to adjust it to the nuclear spin Larmor frequency *ω*_0_, while the matching capacitor provides for matching the impedance of the resonant circuit to the cable and amplifier impedances, that are usually 50 Ω. There are several ways to determine the optimal values of these two capacitances. Commercial NMR spectrometers provide an internal monitoring routine for tuning the probes’ rf circuitry. Most commonly the minimization of the reflected power measured by a reflection bridge or by the use of a Balun circuit with a wobbulator allows this procedure of probe tuning and matching.[11] When this procedure is completed, the entire power delivered by the amplifier is optimally transformed into rf excitation by the coil and there is minimal power reflected to the amplifier, thus avoiding mismatch conditions. This is of particular importance when long and intense rf irradiations are used, for example, for decoupling or transferring magnetization at Hartmann-Hahn conditions.[12] We refer to this well-known procedure as finding the conventional tuning optimum (CTO). Signal-reception is performed by a different part of the circuit, which is activated by a transmit/receive (TR-)switch or through the intrinsic properties of crossed diodes.[11] It was shown previously by noise measurements that, even if this classical tuning is done very accurately, most of the time, it is not optimal with respect to signal reception.[7, 8, 13] One can optimize for signal reception by maximizing the received electronic noise power or, largely equivalent, by optimizing a nuclear spin noise line shape to appear as a symmetrical dip in the noise baseline, as reported previously.[6–8] This alternate tuning procedure, which was called spin noise tuning optimum SNTO[8] can result in higher SNR for NMR signals, when the noise digitization is the limiting quantity.[7, 13]

### 2.2. Three Different Tuning Criteria

**Figure 1:**
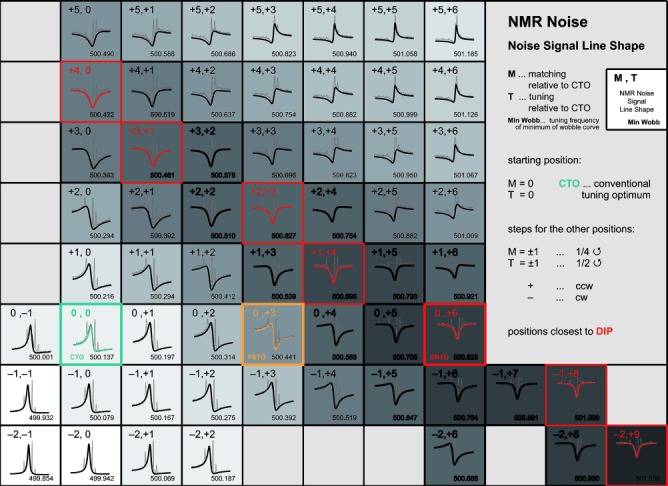
Map of ^1^H spin noise spectra of the sample acetone:acetonitrile:chloroform (1:0.07:0.17)+5 % [D_6_]DMSO by volume, recorded on a 500 MHz Bruker DRX spectrometer equipped with a TXI cryo-probe for different tuning and matching positions. The bold traces are noise signals of acetone, which were deconvolved to avoid interference caused by the narrow superimposed signals of the ^13^C satellites and of acetonitrile. Tuning/matching combinations, where spin noise signals form dips, are indicated by red frames. The FSTO (as found through small-flip angle pulse spectra, see Supporting Information) is highlighted in yellow, while a cyan-colored frame denotes the conventional tuning optimum (CTO), where *γB*_1_ is maximum. Different shades of grey indicate the circuit’s thermal noise power level at each position (darker greys correspond to higher values).

To compare the performance of a cryogenically cooled probe, in which the temperature of the resonant circuit is significantly below the one of the sample, under different tuning and matching conditions, we systematically monitored different parameters on a sample composed such that both high and low spin magnetizations are present. Using a 500 MHz cryo-probe, which had been used in a number of previous investigations,[8, 13–15] line shapes of spin noise signals, rf pulse lengths proportional to 1/*γB*_1_ (with *γ* the gyromagnetic ratio and *B*_1_ the rf amplitude), and frequency shifts 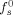 caused by the frequency pushing effect[9, 10] were determined under a variety of conditions. The results are summarized in Figure 1 and in the Supporting Information.

From Figure 1 three special conditions can be defined as follows: (1) *γB*_1_ is maximum at a given rf power delivered by the rf amplifier (CTO condition), (2) the line shape of the spin noise signal is a symmetrical dip of the noise baseline (SNTO condition), and (3) the frequency shift of a high polarization signal is zero, which we call the frequency shift tuning optimum (FSTO condition).

It is noteworthy that, in addition to what was found in previous investigations,[7, 8] there is an infinite number of (*C*_t_, *C*_m_) combinations where the spin noise signal appears as a symmetrical dip line shape (see Figure 1). This degeneracy results from the fact that the SNTO is obtained, whenever the imaginary part of the receiver circuit impedance vanishes, that is, a single physical condition for two adjusted parameters, *C*_t_ and *C*_m_. The position, where the frequency pushing effect is minimal, lies between the CTO and the SNTO condition, at which the signal-to-noise ratio is a maximum. Similarly to the spin noise studies,[7] this mistuning with respect to the SNTO condition can lead to a decrease of the SNR for low receiver gain values (the meaning of “low” depending strongly on the specific hardware used). When high gains can be used to ensure appropriate digitization of noise, no SNR loss is observed.

### 2.3. The Frequency Shift Tuning Optimum

As described previously in ref. [3] and ref. [4], the precessing magnetization *M*_+_ creates the feedback field *ω*_1_ due to the detection circuit:[Disp-formula m1]


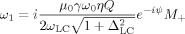
(1)

where *μ*_0_ is the magnetic permeability of free space, *γ* the gyromagnetic ratio, *η* the filling factor and *Q* the apparent quality factor of the loaded resonant circuit with the sample inside. In this equation, both *Δ*_LC_ and *ψ* depend on the mistuning between the Larmor frequency *ω*_0_ and the electronic circuit resonance frequency *ω*_LC_:[Disp-formula m2], [Disp-formula m3]


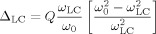
(2)


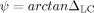
(3)

The angle *ψ* thus represents the deviation of the radiation damping field from quadrature of the precessing magnetization. In the context of the here-reported experiments and more generally for all high-resolution NMR experiments, since the extent of mistuning is in the order of a few hundreds of kHz for Larmor frequencies in the order of several hundreds of MHz, one can simplify the expression of *Δ*_LC_ as:[Disp-formula m4]


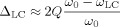
(4)

The so-called frequency pushing effect[5] results from the imaginary part of the feedback field induced by the rf coil not being perfectly orthogonal to the transverse magnetization [Eq. (1)].[3] The frequency pushing 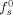 can be described analytically by Equation (5):[10][Disp-formula m5]


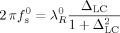
(5)


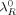
 is the radiation damping rate at perfect tuning (*ω*_LC_=*ω*_0_) and at thermal equilibrium between coil and spin system:[Disp-formula m6]


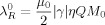
(6)

where *M*_0_ is the longitudinal magnetization at equilibrium with the temperatures *T*_circuit_=*T*_sample_.

The observed resonance full line width at half height for large magnetization, high *Q*, and small flip angle excitation pulses is (*λ_R_*+*λ*_2_)/π, with *λ*_2_ being the transverse self-relaxation rate of the observed species and *λ_R_* the effective radiation damping rate which depends on the tuning conditions:[Disp-formula m7]


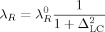
(7)

**Figure 2:**
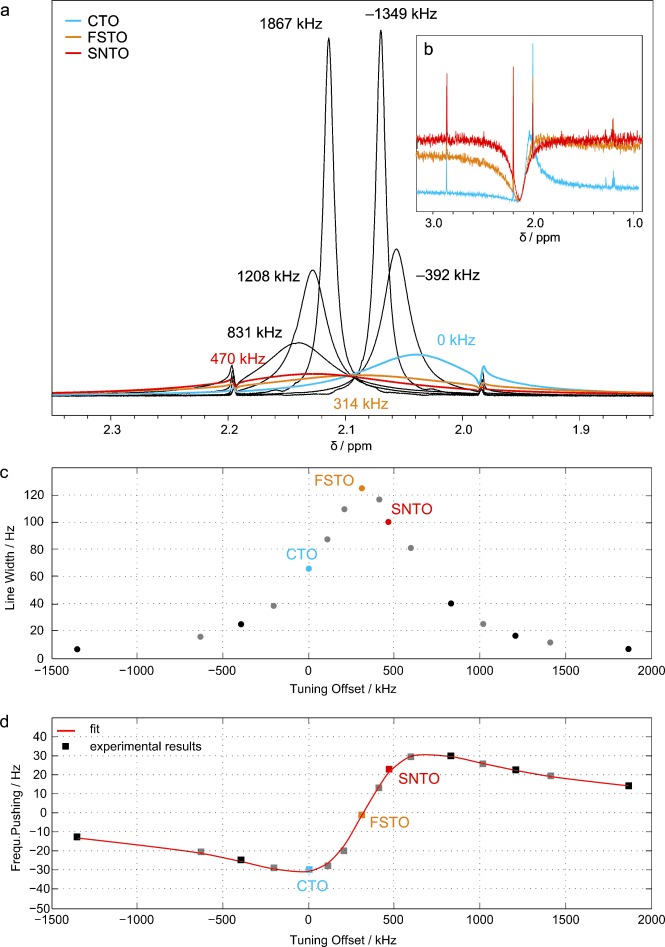
Variation of ^1^H line shapes and resonance frequency shifts of 90 % of acetone in [D_6_]DMSO as a function of the tuning frequency of the electronic detection circuit of a 600 MHz cryo-probe. Colors represent different tuning frequencies ranging from −1.4 MHz up to 1.9 MHz relative to the CTO condition, which is shown in cyan. The FSTO and the SNTO conditions are indicated by yellow and red, respectively. To take into account the variation of detection sensitivity due to the tuning offset, the spectra were normalized to the residual ^1^H_2_O signal (<0.5 % in [D_6_]DMSO), whose concentration was sufficiently low not to be affected by radiation damping. Panel (a) shows small flip angle spectra for various tuning positions with numerical values of the corresponding tuning offsets. The inset panel (b) displays the spin noise spectra only at the three characteristic optimum tuning conditions. The observed line widths and frequency shifts are plotted in the panels (c) and (d), respectively, as functions of the tuning offset relative to the CTO, the experimental points correspond to the spectra shown in (a). The best-fit curve to Equation (5) is drawn in red in panel (d). It is represented by a *Q* of 800, *ω*_FSTO_/2 π= 599.596 MHz and 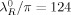 Hz. The observed deviation of the SNTO from the FSTO frequency (*ω*_SNTO_−*ω*_FSTO_)/2 π=−156 kHz is caused by the feedback field. For clarity, the small flip angle spectra corresponding to the grey dots in (c) and (d) are not included in (a).

To assess these effects experimentally in a systematic way, we obtained small flip angle (SFA) pulse and spin noise ^1^H NMR spectra under different tuning conditions on a sample of acetone using a cold probe. The results shown in Figure 2 illustrate the positions and characteristics of the three different tuning optima. In panels a and b SFA and spin noise spectra at different tuning offsets are compared. In Figure 2 c the experimentally determined line widths obtained from the small flip angle spectra of Figure 2 a are plotted as a function of the tuning offset. The line widths are dominated by the radiation damping term and equal to (π*λ_R_*)^−1^ under the experimental conditions of Figure 2. Comparing this plot to the one in Figure 2 d shows that radiation damping is at its maximum at tuning conditions where the frequency pushing effect vanishes, as pointed out earlier by Torchia.[10] Panels c and d of Figure 2 also provide experimental evidence that radiation damping and frequency pushing described by Equations (7) and (5), respectively, are the absorptive and dispersive components of the same effect. Figure 2 also corroborates that on a cryo-probe the difference between the SNTO and FSTO conditions is much larger than the uncertainty of determining the respective tuning conditions.

On all cryo-probes we investigated the FSTO was found between the CTO and the SNTO. Since the three tuning conditions (CTO, SNTO and FSTO) correspond to three different *ω*_LC_ values, we shall in the following denote them as *ω*_CTO_, *ω*_SNTO_ and *ω*_FSTO_, respectively, and similarly we shall use *Δ*_CTO_, *Δ*_SNTO_ and *Δ*_FSTO_ instead of *Δ*_LC_, as appropriate. It is noteworthy and it may be of practical relevance that radiation damping is not at its maximum under SNTO conditions, but coincides with the FSTO [Eqs. (5) and (7)].

In general, the frequency shift as well as the radiation damping rate scale as the longitudinal magnetization *M_z_*, that is 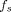
=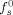
(*M_z_*/*M*_0_) and *λ_R_*=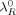
(*M_z_*/*M*_0_). Thus both 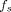 and *λ_R_* depend on the temperature ratio between circuit and sample. This explains the extraordinary tuning behavior of cryogenically cooled probes.[8, 13] By using Equation (4) from ref. [16], the effective circuit temperature can be determined from the concentration dependence of the spin noise signal shape and offset. As a consequence of the frequency pushing effect, the peak position becomes dependent on *M_z_* unless *ω*_FSTO_=*ω*_0_. Therefore, *ω*_FSTO_=*ω*_0_ represents a very favorable situation for solvent suppression, since the solvent peak position does not depend on its *z*-magnetization. It consequently facilitates the fine adjustment of the solvent suppression technique and reduces drift artifacts during long-time experiments, since the frequency pushing on the solvent resonance will not change due to varying solvent magnetization. To be more accurate, a residual longitudinal magnetization dependence of the observed resonance frequency on the order of 1 to 3 Hz remains present (assuming typical high resolution NMR conditions), since, for a nonspherical sample and large magnetization, the exact resonance frequency is affected by long-distance dipolar fields.[4] While in principle homogeneity fluctuations may also cause variations in the Larmor frequency, these will usually be corrected by the field frequency lock, which is much less affected by the radiation field (due to low *γ* of the lock nucleus D). These fluctuations do not lead to appreciable spin-circuit mistuning, because they are in the Hz range, while tuning offsets having an effect on the resonance frequency and line shape are in the kHz range.

Since the frequency shift tuning optimum FSTO can be determined very precisely from a series of single small flip angle pulse experiments (Figure 2) by fitting to Equation (5),[17] or interactively using the isosbestic behavior shown in Figure 2 a we suggest to use it as an alternative tuning optimum, in cases where optimal control of the feedback field properties is sought.

### 2.4. Conventional NMR Probes

We conducted similar studies with conventional high resolution NMR probes (where the resonant circuit and the sample are at the same temperature), exploring tuning settings corresponding to CTO, SNTO and FSTO conditions. The smaller quality factors *Q* of conventional probes reduce the magnitudes of the radiation damping rate [Eq. (6)] and of the frequency pushing effect [Eq. (5)]. Nevertheless by contrast to cold probes, other experimentally accessible degrees of freedom exist. The adaptation of the cable lengths between the TR-switch, the preamplifier and the probe has allowed us to explore the effect of changing the impedance of the transmission line on the observed signals. This feature was previously used for ensuring correct amplifier matching conditions in the SNTO conditions,[6, 7] and was recently employed to study the influence of systematic variation of cable lengths on spin noise line shapes.[18]

We compared spin noise and small flip angle spectra for different cable lengths between the TR-switch and the probe. We observed that the frequency shifts almost vanished at the SNTO conditions: the differences between the FSTO and SNTO conditions were much smaller than in the case of cryo-probes and typically within the measurement uncertainties for defining the SNTO. In the Supporting Information, spin noise spectra acquired with different cable lengths are shown. As also observed on the small-flip angle spectra,[17] the resonance line widths vary. They clearly prove that the signal shapes can be affected by the transmission line impedance beyond the detection coil and associated capacitor. This “remote impedance effect” was further confirmed by noting a small but detectable dependence on the observed SNTO and FSTO positions on the spectrometer receiver gain levels. These sets of experiments illustrate that tuning conditions are influenced by all reception circuit components and not by the probe circuit alone.

### 2.5. Reconciliating FSTO and SNTO?

While it follows from the usage of a T/R-switch or crossed diodes that the CTO can derivate from the reception optimum, the observation of different values for FSTO and SNTO appears counter-intuitive. Indeed assuming, as usual, that the detection circuit can be represented by an RLC circuit resonance, following McCoy and Ernst,[19] the nuclear spin noise spectral density *W*^*U*^ is[Disp-formula m8]:


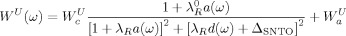
(8)

where 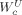 is the spectral density of the resonant electronic circuit, 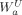 is noise spectral density of other sources such as the preamplifier, and *a*(*ω*) and *d*(*ω*) are the absorptive and dispersive NMR resonance line shapes:[Disp-formula m9], [Disp-formula m10]


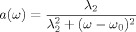
(9)


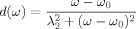
(10)

When perfect tuning conditions (SNTO)[19] are fulfilled, that is:[Disp-formula m11]


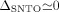
(11)

Equation (8) becomes[Disp-formula m12]:


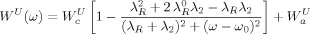
(12)

For the case *T*_circuit_=*T*_sample_ it follows that *λ_R_*=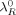 and therefore Equation (12) reduces to a simple Lorentzian-like function, the symmetrical dip line shape observed at the SNTO:[Disp-formula m13]


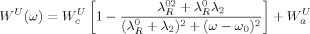
(13)

Comparing Equations (5) and (8) reveals similar dependence of the frequency pushing effect and the spin noise line shape on the tuning frequency offset. The frequency pushing consequently should also vanish at the SNTO. This comparison also illustrates that *Δ*_SNTO_ is the appropriate dimensionless parameter to describe the mistuning. For conventional probes used at room temperature, it is impossible to assess that SNTO conditions are fulfilled, if the deviation is less than about *Δ*_SNTO_=0.05 for a restricted time of spin-noise signal averaging (see Supporting Information). This leads to a potential small dispersive contribution on the spin noise line shape and an ensuing misinterpretation of the resonance frequencies. As a consequence, for these probes SNTO and FSTO conditions are identical within experimental uncertainties. But for cold probes (as in Figure 2), the difference[Disp-formula m14]


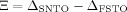
(14)

deviates significantly from zero, that is, a discrepancy between the two *ω*_0_ values determined from Equation (5) and Equation (8), which define the FSTO and SNTO conditions is observed.

This proves that the entire detection circuit cannot be reproduced by a simple RLC resonant circuit model, since it is not sufficient to describe all physical phenomena (i.e. spin noise line shapes and frequency pushing) related to NMR signal detection, as is most evident on cryogenically cooled probes. An improved model should allow the design of probes, where the differences between the three optima vanish.

The set of experiments acquired with room temperature probes described in the Supporting Information corroborates the cable length dependence reported earlier.[18] This shows that the spin dynamics depend on the preamplifier and the TR-switch. Similarly, resonance frequencies and line widths were observed to depend on the receiver gain levels for cold probes by comparing spectra acquired at the same tuning frequency but at different receiver gain levels. Moreover a significant difference was observed when comparing the line widths [Eq. (7)] and the resonance frequencies [Eq. (5)] as functions of the tuning conditions, at constant receiver gain levels, in a procedure similar to that used to obtain Figure 2. The analysis indicates a variation of the extracted apparent *Q* values by 7.5 % over the whole range of receiver gains. This translates into a theoretical variation of the signal-to-noise ratios of 3.5 %.[1] The practical consequences are obviously different since the receiver gain levels have an influence on the digitization noise. Associated with this *Q* variation, the FSTO was observed to vary to an extent of the order of *Δ*_FSTO_=0.05. Even if this receiver gain influence is significant and clearly detectable at the FSTO, it is not sufficient to allow reconciliation of the SNTO and FSTO conditions, since for instance in the case of the cryo-probe in Figure 2 we found a deviation *Ξ*=−0.4.

In further attempts to characterize the difference between FSTO and SNTO, the relative noise levels originating from the preamplifier and from the electronic circuit resonance (coil and sample) were varied. On a conventional probe it became possible to clearly distinguish FSTO and SNTO by decreasing the sample and coil temperatures down to −60 °C (see the Supporting Information). Here, by adjusting the impedance of the transmission line, it was possible to render the three optimum conditions (CTO, SNTO and FSTO) indistinguishable. This is in contrast to a cryo-probe, where the transmission line impedance between coil and preamplifier is invariable. But by increasing the sample conductivity through the addition of salt (Table 1 and Figure S4 in the Supporting Information) we were able to reduce the difference *Ξ* significantly. Apparently the increased salt concentration caused a higher total noise level from the receiving coil assembly making the situation more comparable to a conventional probe. From Table 1 it is evident that, while the apparent difference of the two tuning frequencies increases with the ionic strength, the dimensionless parameter *Ξ* decreases. These two sets of experiments substantiate the key importance of electronic components beyond the probe on the manifestation of different SNTO, FSTO and CTO conditions.

**Table 1:** Differences between the FSTO and SNTO frequencies and corresponding mistuning parameters *Ξ* [Eq. (14)] as a function of the salt concentration *c*_NaCl_ in a H_2_O : D_2_O=1:1 sample on a 700 MHz TCI cryo-probe (see Supporting Information)

*c*_NaCl_ [mmol L^−1^]	(*ω*_SNTO_−*ω*_FSTO_)/2 π [kHz]^[a]^	*Ξ*^[a]^
0	−236±19	−0.42±0.05
100	−235±29	−0.20±0.03
200	−318±38	−0.20±0.03
300	−335±28	−0.15±0.02
500	−433±81	−0.15±0.04

[a] The reported uncertainties correspond to fitting errors. Since FSTO and SNTO frequency values do not conform to the theoretical model predictions, some systematic errors are present.

## 3. Conclusions

Our results from a plethora of different probes (we have run similar experiments, albeit not as systematic and thorough as the ones reported here, on at least seven different probes and spectrometers) consistently show that, there are three different “optimal” tuning and matching combinations on cold probes. Usually probes are optimized for optimum pulse performance at the CTO. The two other optima (SNTO and FSTO) are most often indistinguishable on conventional probes at room temperature, but they can be significantly different at lower temperatures and in particular for cryogenically cooled probes.

Each of the tuning optima provides different benefits depending on the requirements of the sample and the type of experiment. The conventional tuning optimum CTO allows for the shortest pulse lengths. It should be used whenever excitation efficiency is most important. At the frequency shift tuning optimum, FSTO, radiation damping is at its maximum and there is no frequency pushing effect, which means that the feedback field is in perfect quadrature to the transverse magnetization. Using the FSTO is advantageous for solvent (water) signal suppression experiments, since the solvent resonance position is not shifting as a function of total *z*-polarization. It thus provides conditions for more stable solvent suppression even in cases where the homogeneity fluctuates during an experiment or where the solvent concentration changes, for example, through evaporation. At the SNTO we find optimum reception conditions, that provide signal-to-noise gains for small receiver gains. It is also the most favorable condition for spin noise imaging[14] and spectroscopy, in particular the recently introduced 2D variant.[20]

Comparing experimental results to theoretical predictions it becomes clear that the simple RLC model for describing the NMR electronic probe circuit cannot explain these differences. The FSTO and SNTO conditions are dependent on the input impedance of the preamplifier, which varies with the receiver gain, and on the cable length between the probe and the preamplifier, as well as on the conductivity of the sample. Inclusion of additional probe components including the transmission line, the preamplifier and their temperatures will therefore be required in future theoretical models.

## Experimental Section

### Determination of the SNTO and the Tuning-Matching Map

The data shown in Figure 1 for the 500 MHz cryo-probe were obtained on a 2003 model Bruker TXI cryo-probe connected to a DRX console. To minimize the impact of electronic noise generated by the pulse amplifier and other spectrometer hardware the main supply of the proton pulse amplifier was turned off and the BNC-connector to the proton pre-amplifier was replaced by a 50 Ω terminator while acquiring spin noise spectra. While it is straightforward to trace the actual tuning position as the frequency offset of the minimum of the “wobble” curve, there is no direct way to map the matching position. Therefore adjusting tuning and matching reproducibly is rather difficult. In the end, we used the following rather simplistic approach on a probe with manual tuning and matching controls, which turned out to be effective and sufficiently reproducible. The amount of rotation was controlled by aligning marks at the bottom of the tuning and matching screws with a cross-hair mark drawn on a mirror placed below the probe. To map the signal response versus tuning and matching, the tuning screw was rotated in half turn increments and the matching adjusted in steps of quarter turns.

At each combination of tuning and matching positions thus adjusted, a spin noise spectrum was recorded using the block wise pseudo-2D method described previously.[8] Before changing the tuning and matching settings the reference pulse spectra were acquired (the amplifier cables had to be de- and reconnected for each of these steps). The noise spectra were processed and the thermal noise level at each position was determined using short Matlab programs.

### Determination of the FSTO

For Figure 2 experimental spectra were acquired on a Bruker Avance II 600 spectrometer equipped with a TXI cryo-probe built in 2008. The small flip angle spectra were acquired with a 0.33 μs pulse at 3.8 W (corresponding to a 3° flip angle on resonance). The sample consisted of acetone 90 % in [D_6_]DMSO. The observed signal was the acetone one. The frequency shift curve in Figure 2 d was obtained by plotting the chemical shift at the maximum of the NMR resonance signal exhibiting radiation damping against the tuning offset. The tuning offset was defined relative to the CTO. The *C*_t_ and *C*_m_ capacitances were optimized at a given offset to the lowest wobble curve by matching at 50 Ω. Note that in all experiments used for the study the reproducibility of the setting of the tuning position was limited by the mechanical properties of the probe assemblies to approximately ±40 kHz. However, the offsets could be determined with a higher accuracy through the fitting procedures used. In addition to the small flip angle pulse experiments a pre-saturation pulse experiment was performed to attenuate radiation damping and obtain the reference shift. The latter can also be determined as the median of the spinning side bands, when spinning the sample slowly (ca. 10 Hz) or from the isosbestic intersection of the SFA spectra (Figure 2 a). Apart from that, the frequency pushing is zero at the inflection point of the frequency pushing curve [Eq. (5)] and Figure 2 d determined by curve fitting.

Using the frequency shifts and the line widths of the signals at each tuning position, it is possible to estimate the apparent quality factor *Q* of the probe without resorting to external hardware (for example, a network analyzer).[10] Alternatively the entire curve was fitted to Equation (5) using the experimental (maximum) line width of the signal at zero frequency shift. The maximum observed line width of the signal from a sample containing 90 % of acetone in [D_6_]DMSO, was 124 Hz. The fitted apparent circuit quality factor *Q* was ∼800. It should be noted that the *Q*-values determined in this paper refer to the entire circuit as opposed to the coil only.

For Table 1 frequency shifts and tuning positions obtained on a 700 MHz TCI Bruker Avance III cryo-probe system (manufactured in 2011) were used to fit to Equation (5) to obtain the zero frequency shift and *Q*.

Conventional probe experiments were run on a Bruker Avance 500 spectrometer with a 5 mm BBI probe on an acetonitrile sample with 10 μL of [D_6_]DMSO used for field frequency locking. Series of small flip angle ^1^H spectra were acquired for different cable lengths and different tuning positions (varying CTO from 498.1 to 501.1 MHz). From tuning positions close to that leading to pure in-phase spin noise spectra, SNTO conditions were determined by real-time monitoring of the electronic noise level while changing the tuning capacitor *C*_t_.[21] Spin noise spectra were acquired as a pseudo 2D map with very long acquisition times. The time domain data were split and Fourier transformed using a sliding window approach during the processing.[4, 21] Analogous procedures (frequency shift curves and comparison of the spin noise line shapes) were used for exploring effects of low temperatures or of changing the receiver gain levels.

## Supporting Information

As a service to our authors and readers, this journal provides supporting information supplied by the authors. Such materials are peer reviewed and may be re-organized for online delivery, but are not copy-edited or typeset. Technical support issues arising from supporting information (other than missing files) should be addressed to the authors.

miscellaneous_information
