# Supplementary material for: On the Tuning of High-Resolution NMR Probes
Source: Chemphyschem. 2014 Sep 11;15(16):3639–45. doi: 10.1002/cphc.201402236 (PMC4501310; doi:10.1002/cphc.201402236)
Supplement: Supplementary file 1 — miscellaneous_information [file cphc0015-3639-sd1.pdf]

CHEMPHYSCHEM

Supporting Information

© Copyright Wiley-VCH Verlag GmbH & Co. KGaA, 69451 Weinheim, 2014

On the Tuning of High-Resolution NMR Probes

Maria Theresia Pöschko,[a] Judith Schlagnitweit,[a, c] Gaspard Huber,[b] Martin Nausner,[a] Michaela Horničáková,[a, d] Hervé Desvaux,*[b] and Norbert Müller*[a]

cphc_201402236_sm_miscellaneous_information.pdf

1 Tuning and matching map

For Figure 1 frequency shifts, noise signal lineshapes, thermal noise and frequency shifts in small flip angle pulse spectra were acquired for all tuning and matching combinations shown. The 90° pulse was calibrated for each set of tuning/matching values by using a 40 dB attenuator in the receiver line according to reference [1]. In order to obtain a reasonably high receiver gain without producing an ADC overflow, noise in spite of the high proton concentration, and to avoid that the noise is dominated by the digitizer, a pulse with flip angle of 2° was used in the pulse spectra used to determine frequency shifts and S/N ratios. The same sample (acetone : acetonitrile : chloroform (1 : 0.07 : 0.17) + 5 % DMSO-d_6 by volume) was used as for the noise experiments. For the signal-to-noise measurements the chloroform signal was used. Additional data to the ones shown in Fig. 1 were also recorded at each tuning/matching position. Figure S1 shows the B_1 field strengths relative to the CTO, signal to noise ratios and frequency shifts for each tuning and matching position. In addition, color coded small maps are shown, which summarize the trends relative to the values obtained at the CTO (shown in white). The positions, for which the shortest pulse lengths were found, are colored pink. On the central small map the darkest square indicates the highest signal to noise ratio obtained. Note that this position does not coincide with the CTO. The third small map illustrates the deviation of the signal maximum (the frequency shift) from the reference chemical shift, the lighter the color, the smaller the deviation. Note that the smallest deviation was found at exactly the same tuning/matching position as the signal to noise maximum.

The position of the S/N maximum is in apparent contradiction to previous results[2] for 2 mM sucrose dissolved in 90% H_2O/10% D_2O, where the maximum signal and maximum signal-to-noise was found at the SNTO position (starting from CTO by detuning and matching to the base line until the spin noise line shape was a pure dip) using the same probe and spectrometer. Therefore the relevant part of the tuning/matching map was repeated using this sample. For the signal-to-noise determination the intensity of the anomeric doublet was read out and compared to the noise (region: 7–9 ppm) using Matlab. In this case the investigated signal is close to the water signal. Therefore the pulse experiments were carried out with presaturating the water signal, as is usual. Figure S2 shows the result of these measurements for different tuning and matching positions[1].

This clearly shows that with this sample the maximum S/N is at the SNTO, confirming the previous results. It can be seen that the optimum positions obtained on the sucrose sample differ from the results obtained with the acetone/acetonitrile sample.

It was suspected that partial overlap between the solvent signal and the anomeric doublet, may cause this deviation. Therefore the experiments were repeated using 10 mM acetaldehyde in 90% H_2O/10% D_2O. Pulse experiments were carried out with attenuators and without, using presaturation, for comparison. The results obtained using this sample resemble those obtained with the acetone/acetonitrile sample. The position where the signal-to-noise ratio is a maximum coincides with the tuning/matching position where the frequency shift is at a minimum, for both, the presaturation and the small flip angle spectra. However in the same spectra, comparing a contamination signal at 3.9 ppm, i.e. much closer to the water chemical shift and therefore comparable to the

[1]It has to be noted that the quality of presaturation varies with different tuning/matching settings. Since the same, reasonably high receiver gain could be used for all the experiments without producing an ADC overflow, this fact is neglected.

	+5, 0<br>0.526<br>2189.5<br>–19.43	+5,+1<br>0.443<br>2035.9<br>–19.73	+5,+2<br>0.369<br>1889.3<br>–18.98	+5,+3<br>0.312<br>1611.4<br>–17.68	+5,+4<br>0.268<br>1392.5<br>–16.23	+5,+5<br>0.235<br>1158.7<br>–14.68	+5,+6<br>0.208<br>1088.1<br>–13.18			
	+4, 0<br>0.614<br>2348.2<br>–15.58	+4,+1<br>0.532<br>2347.7<br>–18.13	+4,+2<br>0.443<br>2252.3<br>–18.73	+4,+3<br>0.371<br>1859.8<br>–18.08	+4,+4<br>0.317<br>1755.0<br>–16.78	+4,+5<br>0.274<br>1444.1<br>–15.23	+4,+6<br>0.242<br>1359.3<br>–13.93			
	+3, 0<br>0.757<br>2424.5<br>–5.48	+3,+1<br>0.640<br>2398.1<br>–13.73	+3,+2<br>0.524<br>2305.4<br>–17.03	+3,+3<br>0.435<br>2170.1<br>–17.58	+3,+4<br>0.368<br>1890.1<br>–16.78	+3,+5<br>0.320<br>1749.5<br>–15.93	+3,+6<br>0.281<br>1602.3<br>–14.3285			
	+2, 0<br>0.876<br>2438.4<br>+6.53	+2,+1<br>0.747<br>2375.0<br>–4.3765	+2,+2<br>0.619<br>2480.3<br>–12.28	+2,+3<br>0.509<br>2285.1<br>–15.53	+2,+4<br>0.435<br>2176.8<br>–16.13	+2,+5<br>0.369<br>1894.5<br>–15.68	+2,+6<br>0.323<br>1739.1<br>–14.68			
	+1, 0<br>0.979<br>2554.6<br>+11.53	+1,+1<br>0.892<br>2534.2<br>+7.43	+1,+2<br>0.755<br>2431.5<br>–1.33	+1,+3<br>0.621<br>2427.6<br>–8.98	+1,+4<br>0.475<br>2319.7<br>–14.63	+1,+5<br>0.440<br>2218.2<br>–14.53	+1,+6<br>0.381<br>2056.8<br>-14.48			
0 ,–1<br>0.968<br>2424.9<br>+14.63	0 , 0<br>1.000<br>2557.0<br>+13.23<br>CTO	0 ,+1<br>0.986<br>2712.6<br>+11.43	0 ,+2<br>0.885<br>2748.1<br>+7.03	0 ,+3<br>0.741<br>2853.2<br>+0.07<br>SINO MAX	0 ,+4<br>0.617<br>2590.8<br>–6.53	0 ,+5<br>0.522<br>2550.3<br>–10.48	0 ,+6<br>0.452<br>2369.1<br>–12.28			
–1,–1<br>0.909<br>2346.6<br>+13.128	–1, 0<br>0.994<br>2469.4<br>+12.33	–1,+1<br>1.013<br>2506.3<br>+11.18<br>$\gamma B_1/2\pi$ MAX	–1,+2<br>0.936<br>2676.7<br>+8.58	–1,+3<br>0.816<br>2647.7<br>+5.33	–1,+4<br>0.690<br>2657.3<br>–1.23	–1,+5<br>0.589<br>2545.1<br>–5.78	–1,+6<br>0.506<br>2363.3<br>–9.18	–1,+7<br>0.438<br>2214.8<br>–10.83	–1,+8<br>0.396<br>2183.2<br>–11.23	
–2,–1<br>0.841<br>2237.0<br>+11.48	–2, 0<br>0.932<br>2374.9<br>+11.38	–2,+1<br>1.001<br>2389.4<br>+10.83	–2,+2<br>0.990<br>2628.6<br>+9.53				–2,+6<br>0.575<br>2520.1<br>–4.38		–2,+8<br>0.441<br>2284.3<br>–8.58	–2,+9<br>0.393<br>2325.7<br>–9.43

Pulse Spectra:

B_1 rel, SINO, shift

$\gamma B_1/2\pi$ rel. to CTO
signal to noise
shift [Hz]

M , T
B_1 rel
SINO
shift

$\gamma B_1/2\pi$ rel. to CTO:

calibrated using 40dB attenuator according to [X]

0.208 - 1.013

signal to noise:

signal intensity of $CHCl_3$
noise region: 9 - 14 ppm

1088.1 - 2853.2

shift:

shift of the acetone peak relative to the center of the ^{13}C-satellites

–19.73 - +14.63

because of the high proton concentration a pulse with flip angle of 2° was used

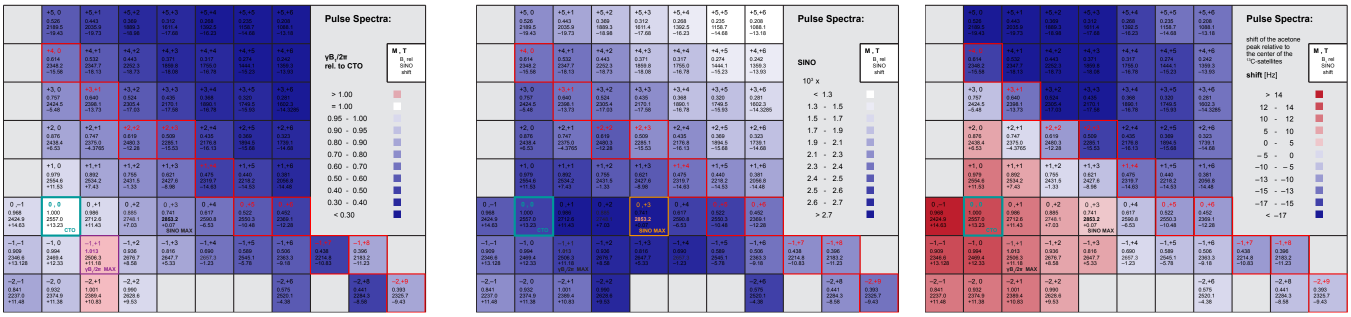

Figure S1: Comparison of γB_1, signal-to-noise and frequency shift values for each tuning/matching position. The three small maps are colored to show the trends separately for γB_1, frequency shifts and S/N.

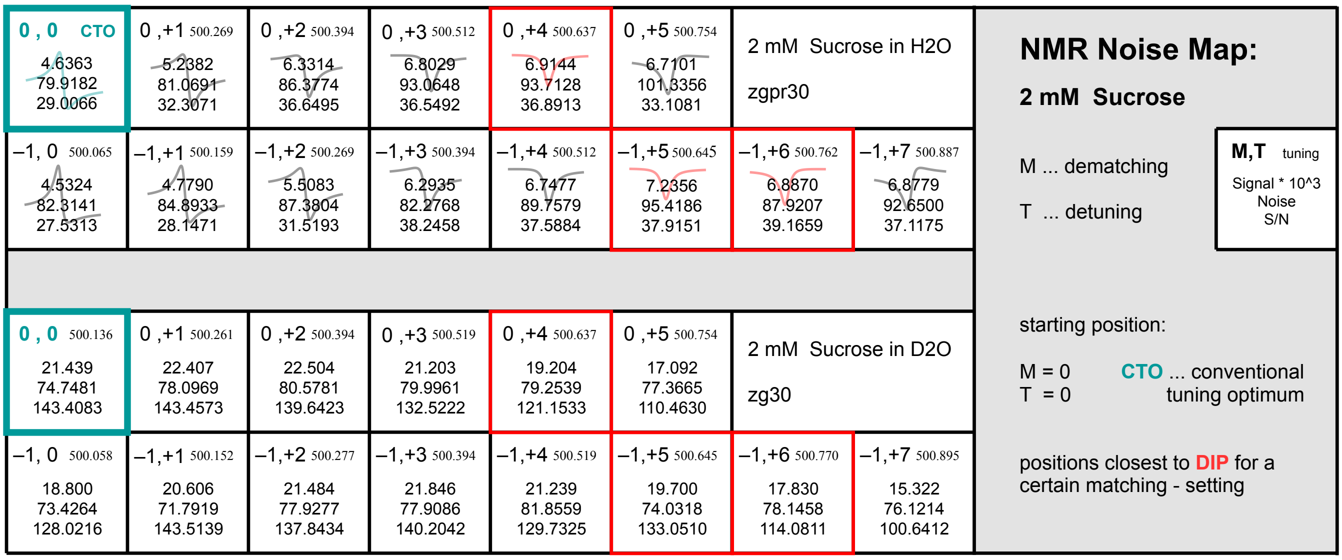

Figure S2: Comparison of signal, noise and signal-to-noise ratios of the anomeric doublet (at 5.3 ppm) for different tuning- and two different matching settings. The results for 2 mM sucrose in H_2O (upper part) and in D_2O (lower part) are shown. Noise line shapes are shown in the upper part.

sucrose anomeric doublet, the signal-to-noise maximum is found at the SNTO. It appears, that the position of the signal-to-noise maximum is different for signals close to a much stronger resonance signal, a finding which is corroborated by recent observations in small flip angle spectra.[3]

2 Experiments with conventional NMR probes

The exploration of differences between CTO, SNTO and FSTO conditions on conventional probes was performed for several cable lengths between the TR-switch and the probe. Experiments were run on a Bruker Avance II spectrometer (11.7 T) equipped with a 5 mm BBI probe on an acetonitrile sample with 10 μL of deuterated DMSO used for field frequency locking. We used a real-time software for determining the SNTO condition from tuning frequencies close to that leading to pure in-phase spin-noise spectra. The optimal conditions were found by monitoring the circuit noise level while changing the tuning capacitor C_T and trying to center the noise level due to the electronic circuit at the Larmor frequency.[5] Nuclear spin-noise spectra were then acquired as a pseudo 2D map with very long acquisition (43 s). The final resolution was defined during the processing. The raw time-domain noise data were split in a sliding window way.[4,5] The split raw data lead to 0.8 Hz spectral resolution zero-filled to 0.4 Hz. Quantitative analysis were performed on these spectra. For clarity the two spectra shown in Fig. S3 were further filtered by a Savitzky-Golay smoothing filter (fourth order polynomial, 25 points).[6]

On Fig. S3, two spin-noise spectra acquired for two different cable lengths are shown on the experimentally found SNTO conditions. One can notice the difference of resonance frequency but also of line-width illustrating the influence of the reception line on the spin dynamics. Even if a

difference of resonance frequency can be detected on these two spectra, when comparing to the FSTO conditions as determined by monitoring the ^{1}H acetonitrile resonance frequency as a function of the tuning frequency, it was impossible to distinguish FSTO and SNTO values, due to experimental uncertainties.

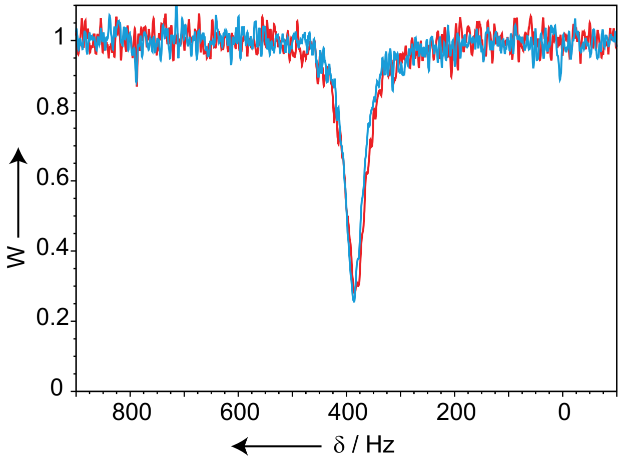

Figure S3: Spin-noise spectra acquired on a room temperature probe on an acetonitrile solution for two different cable length in the SNTO conditions. The two spectra are normalized at the same circuit noise level. As confirmed by numerical fitting the spin-noise spectra to Eq. (13) of the main text, the two line-widths and the two resonance frequencies are slightly different.

Figure S4 illustrates this difficulty of defining very precisely and accurately the frequency of SNTO condition. The rough determination of the SNTO frequency was obtained by electronic measurements, one was searching for the capacitor value for which the frequency of the maximum value of the bell-shape curve of circuit noise was equal to the Larmor frequency. Obviously this curve was affected by noise fluctuations, which amplitude depended on duration of signal averaging. The typical width of the bell-shape curve was $\omega/2Q$ that was about 750 kHz; a rough estimation of the uncertainty on the SNTO frequency was about one tenth of this width, i.e. 75 kHz. A more precise definition could be obtained by looking to nuclear spin-noise resonance, and finding conditions where it fitted the Lorentzian shape (Eq. (13) of the main text). It remained that noise fluctuations were still present and the uncertainty on the SNTO condition defined through the parameter Δ_{SNTO} was on the order of 0.05 for the here used duration of signal averaging (7 minutes). At such a level of precision it was impossible to distinguish SNTO and FSTO conditions with a conventional probe at room temperature.

The situation was different for a low-temperature experiment (sample and coil at $-60°$C). The low temperature induced a lower resistance of the coil and thus a variation of the relative weight of the preamplifier and coil noise sources. After having chosen a length of the transmission line such that the CTO frequency ω_{CTO} was significantly different from the SNTO frequency ω_{SNTO}, the difference between FSTO and SNTO ($\Xi = \Delta_{\mathrm{SNTO}} - \Delta_{\mathrm{FSTO}}$) became significant and clearly detectable. This is illustrated in Fig. S5. In this run of experiments several spin-noise spectra were acquired for tuning frequencies close to the SNTO and FSTO. One can notice that for tuning frequency corresponding

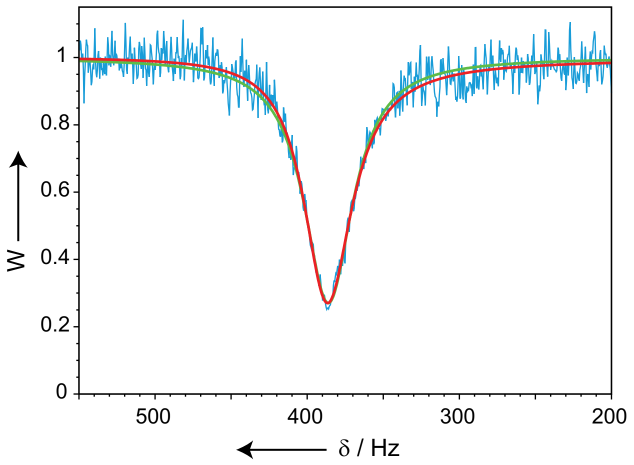

Figure S4: Unfiltered spin-noise spectrum shown in Fig. S3 (blue line) and best-fit theoretical curve to Eq. (8) assuming perfect tuning conditions Δ_{SNTO} (green curve) or without this assumption (red curve). In this last condition, the extracted parameter Δ_{SNTO} was found equal to -0.045 ± 0.005. Such a mistuning would lead to a frequency shift of 1.6 ± 0.2 Hz. In comparison the difference of fitted resonance frequencies of the blue and green spectra of Fig. S4 was 3.4 Hz and the corresponding difference measured on small flip angle spectra was found equal to 2.4 Hz (fitting uncertainties were typically on the order of 0.2 Hz and consequently small when compared to biases). This illustrates that within experimental uncertainties FSTO and SNTO cannot be distinguished for conventional probes at room temperature due to lack of precision on SNTO.

to the FSTO as determined by fitting the frequency pushing effect, a phase-distorted Lorentzian curve was obtained on the spin-noise spectra, while a pure in-phase Lorentzian spin-noise spectrum (SNTO condition) was obtained for a tuning frequency different from that where frequency pushing vanished.

3 Influence of salt on the SNTO and FSTO frequencies on a cold probe

Complementing the low temperature experiments, the deviation of the FSTO from the SNTO (Table 1) was determined for different salt concentrations on a cryo-probe. The frequency pushing effect decreases with increasing salt concentration because of the lower Q. Simultaneously the mistuning parameter Ξ also decreases (see Table 1). This corroborates the decreasing impact of mistuning on frequency pushing and dispersive spin noise line-shapes although the absolute difference between the SNTO and FSTO frequencies increases.

In order to fit the experimental spin noise spectra (Fig. S6) to Eq. (8) a T_{circuit} value of 57 K was used. This value was determined for the same probe by fitting the concentration dependence of spin noise spectra in Ref. [7] but was not explicitly given there. This temperature lies between the nominal temperatures of the observe coil and of the preamplifier, since owning to the construction of cryogenically cooled probes a temperature gradient exists across the probe's resonant circuit and transmission lines. T_{sample} was 298 K. The noise spectra were acquired in 512 noise blocks with 1.56 s each, the final resolution was set to 3 Hz during the sliding window processing.[4,5]

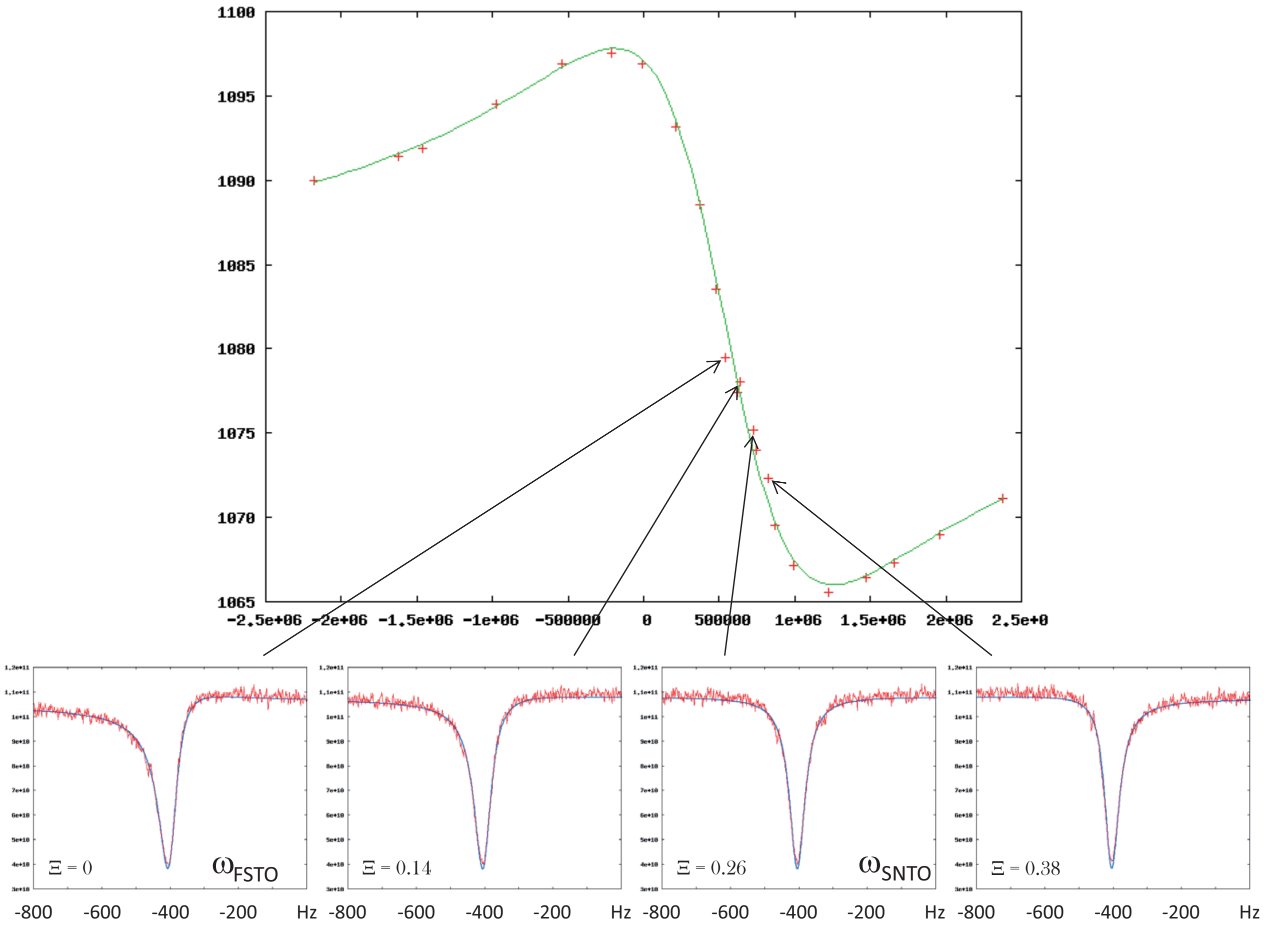

Figure S5: Frequency pushing curve obtained on a conventional probe with a low temperature sample and coil (−60°C). The offset tuning frequencies (in Hz) are defined relative to Larmor frequency. The best-fit theoretical curve to Eq. (5) is superimposed. Four spin-noise spectra acquired for four frequency tuning conditions are shown (acquisition time 30 minutes). For the FSTO condition (spectrum on the left), the spin-noise resonance was not of pure in-phase Lorentzian shape. While the tuning frequency increased as characterized by the Δ factor where the reference was defined for the FSTO condition, the spin-noise resonance tended to an in-phase Lorentzian shape, the point defining the SNTO condition. This condition was very close to the third spectrum, 188 kHz apart from the first spectra in FSTO condition (this offset corresponds to $\Xi = 0.25$). This illustrates that FSTO and SNTO can significantly be distinguished for conventional probes at low temperature.

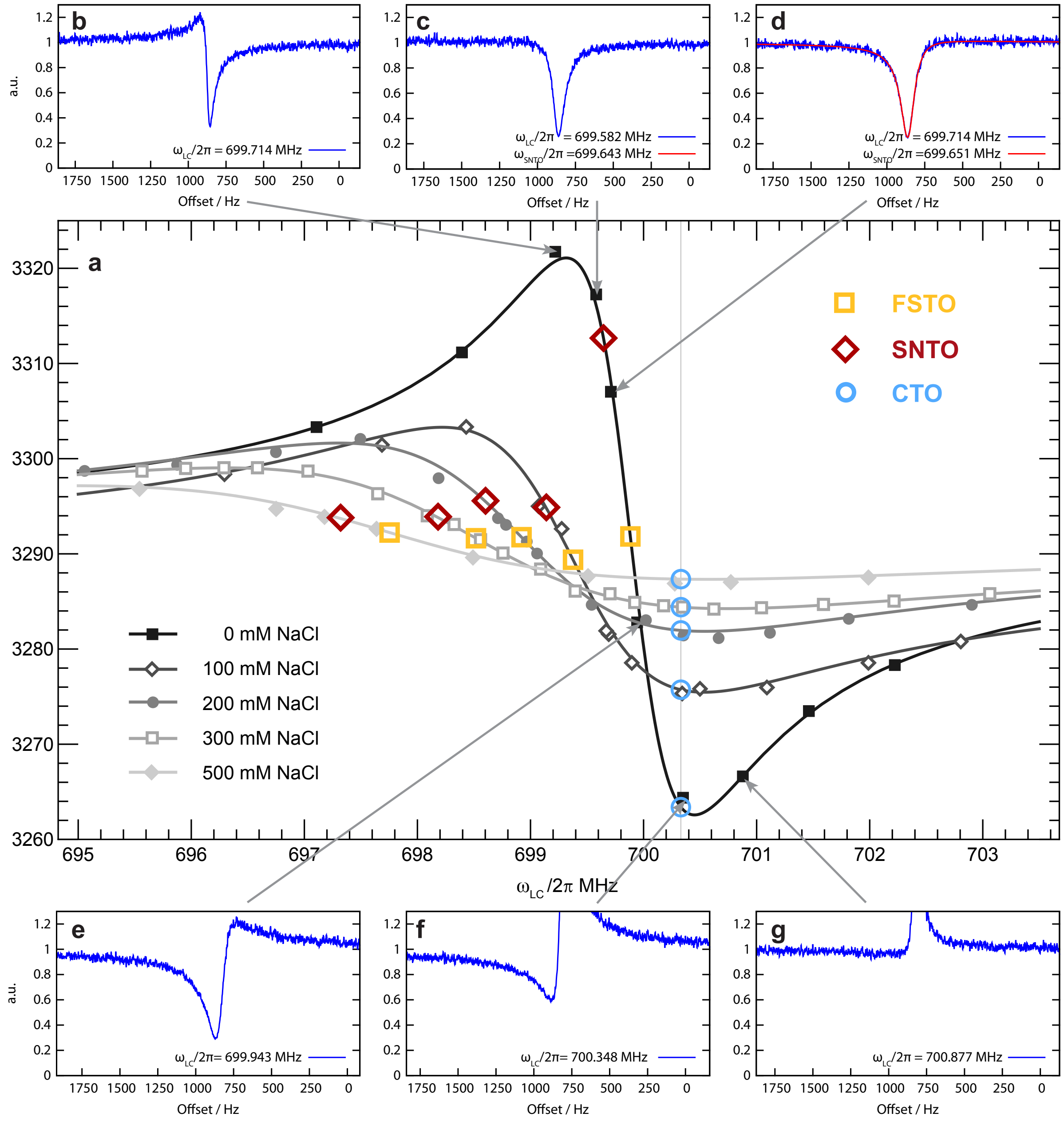

Figure S6: a) Resonance frequency versus tuning position curves of the water signal in samples of $H_2O:D_2O=1:1$ at different NaCl concentrations recorded on a 700 MHz TCI cryo-probe. Experimental data (plot markers) and fit (lines) are superimposed for each series. The FSTO frequencies ω_{FSTO} (yellow) were determined by fitting the experimental points to Eq. (5). The positions of the SNTOs (red) on the individual curves were determined by fitting spin noise spectra at the adjacent tuning positions to Eq. (8), weighting them according to $1/(1 + (\Delta_{\text{SNTO}})^2)$ and substituting the resulting frequency into Eq. (5).
b–g) Spin noise spectra (blue) at six different tuning positions ω_{LC} for the 0 mM NaCl series. Panels c and d include fits (red) to Eq. (8), the resulting ω_{SNTO} is given in the legend of each spectrum.

References

[1] P.S.C. Wu, G. Otting, *J. Magn. Reson.* **2005**, *176*, 115–119.

[2] J. Schlagnitweit, *Increasing the Efficiency of NMR by Multiplex Data Acquisition and Alternative Probe Tuning*, Dissertation, Johannes Kepler University **2011**, pp. 55,72.

[3] J. Schlagnitweit, S. W. Morgan, M. Nausner, N. Müller, H. Desvaux, *ChemPhysChem* **2012**, *13*, 482–487.

[4] H. Desvaux, *Prog. NMR Spectrosc.* **2013**, *70*, 50–71.

[5] H. Desvaux, D. J. Y. Marion, G. Huber, P. Berthault, *Angew. Chem. Int. Ed.* **2009**, *48*, 4341-4343.

[6] A. Savitzky, M. J. E. Golay, *Anal. Chem.* **1964**, *36*, 1627.

[7] J. Schlagnitweit, N. Müller, *J. Magn. Reson.* **2012**, *224*, 78–81.
